# Recurrent Nonischemic Left Ventricular Aneurysms: A Cardiac MRI–Driven Diagnosis in a *DES* Variant Carrier

**DOI:** 10.1016/j.jaccas.2026.109663

**Published:** 2026-08-05

**Authors:** Nathalia Prata Garcia, Diego Forti, Leticia Araujo Tassine Penatti, Henrique Turin Moreira, Benedito Carlos Maciel, André Schmidt, Gustavo Jardim Volpe

**Affiliations:** a Hospital das Clínicas da Faculdade de Medicina de Ribeirão Preto, University of São Paulo, Ribeirão Preto, São Paulo, Brazil; b University of São Paulo, Ribeirão Preto, São Paulo, Brazil

**Keywords:** cardiac magnetic resonance, chest pain, computed tomography, genetic disorders, ventricular tachycardia

## Abstract

**Background:**

Cardiac magnetic resonance (CMR) is the gold-standard technique for characterizing nonischemic structural cardiomyopathies, offering tissue characterization and precise localization of myocardial injury.

**Case Summary:**

A 24-year-old man presented with recurrent chest discomfort and tachyarrhythmias. Initial CMR revealed an apical left ventricular aneurysm with imaging features consistent with a true aneurysm and normal coronary arteries, successfully treated surgically. Five years later, recurrent ventricular tachycardia prompted re-evaluation; CMR identified a new inferolateral basal aneurysm in a region previously normal on postoperative imaging. Coronary computed tomography angiography again showed normal epicardial coronary arteries. Surgical patch repair and implantable cardioverter-defibrillator placement were performed. Genetic testing revealed a pathogenic desmin (*DES*) c.785A>T (p.Glu262Val) variant and additional variant of unknown significance in *VCL* and *CDH2*.

**Discussion:**

The identification of a pathogenic *DES* variant suggests a potential underlying susceptibility to structural remodeling, although causality cannot be confirmed.

**Take-Home Messages:**

CMR was crucial in identifying sequential, anatomically distinct left ventricular aneurysms. The combination of recurrent nonischemic aneurysms and a pathogenic *DES* variant suggests a possible cytoskeletal genetic cardiomyopathy with arrhythmic risk.

## Visual Summary

**Figure:**
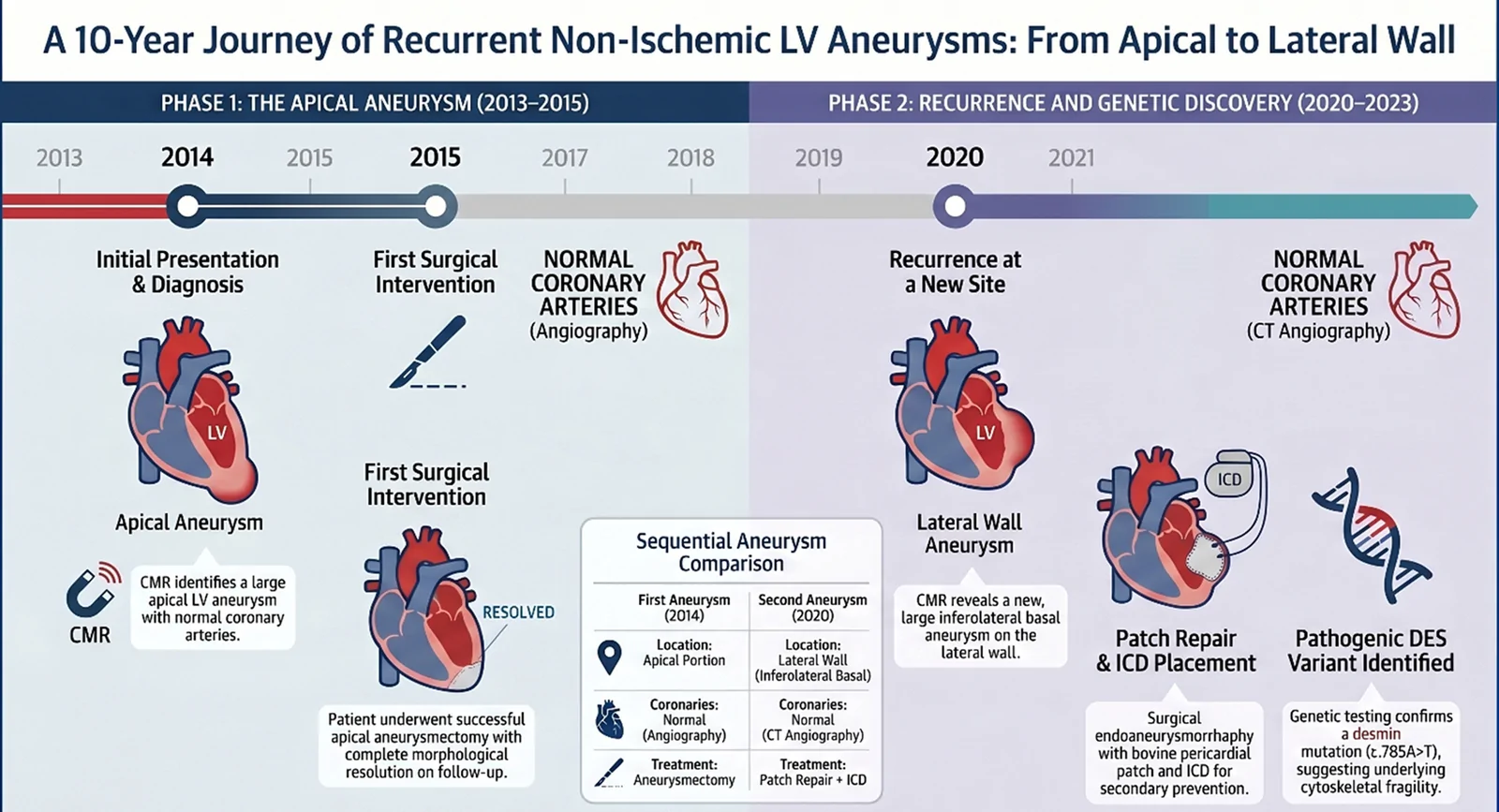

## History of Presentation

A 31-year-old man presented with recurrent episodes of rapid palpitations and presyncope. He described intermittent, nonexertional, oppressive chest discomfort radiating to the neck. Physical examination revealed a sustained, laterally displaced apical impulse with no murmurs. He was hemodynamically stable throughout. The electrocardiogram demonstrated sustained monomorphic ventricular tachycardia (VT), which was successfully terminated with intravenous amiodarone. He was then referred to a tertiary hospital for implantable cardioverter-defibrillator (ICD) placement and further investigation.

## Past Medical History

At age 23, the patient had experienced episodes of chest discomfort and tachyarrhythmias, requiring amiodarone-mediated cardioversion. Evaluation at an outside institution included cardiac magnetic resonance (CMR), which identified a large apical aneurysm (Video 1), and invasive coronary angiography, which demonstrated normal epicardial coronary arteries. He subsequently underwent apical aneurysmectomy at that institution in 2015, with excellent recovery and normalization of apical morphology on postoperative CMR (Figure 1, Video 2). The original angiographic cine recordings and operative report from that procedure were unavailable for review. He remained asymptomatic for nearly 5 years.

**Figure 1:**
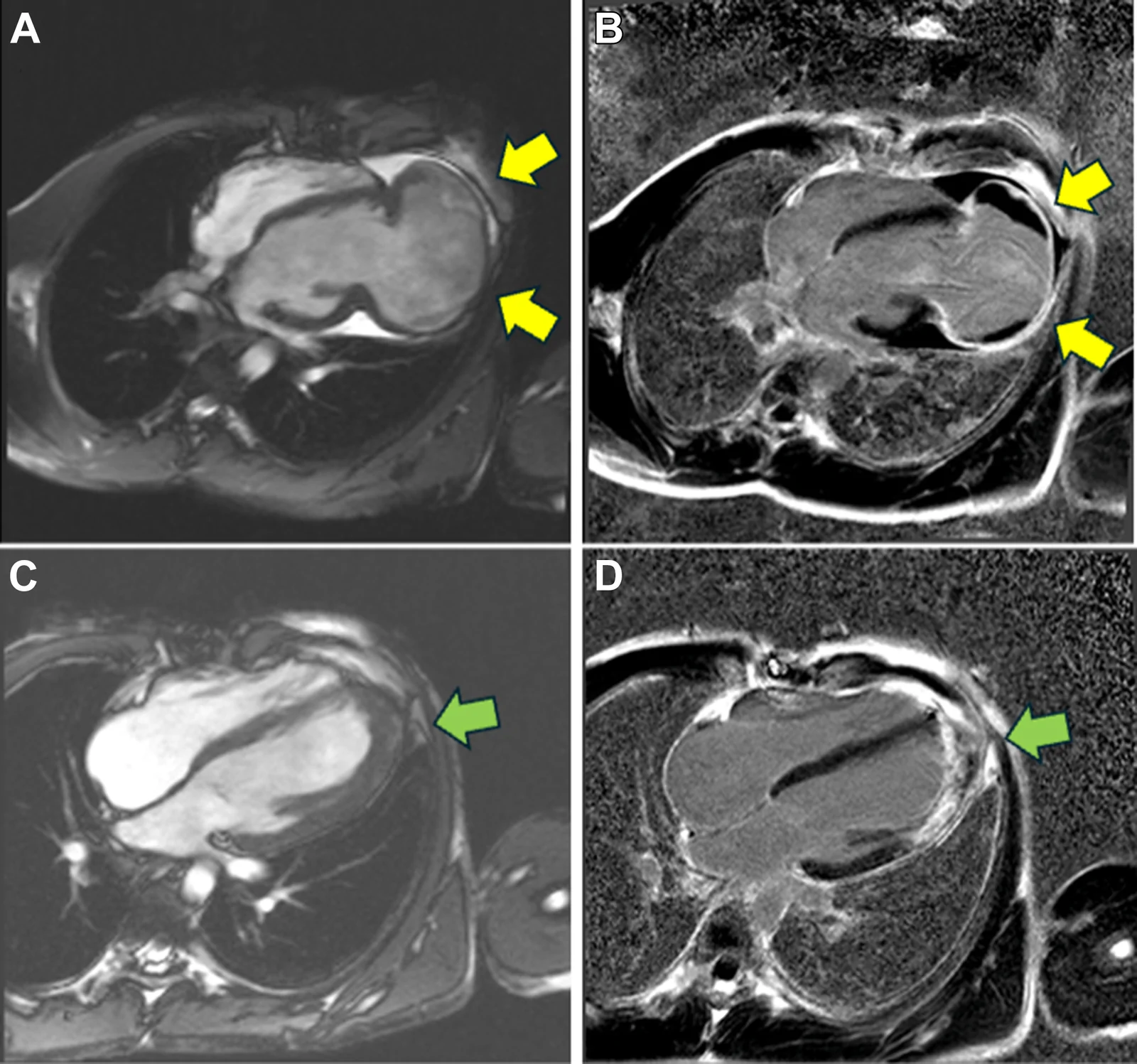
First Aneurysm (Cine SSFP and LGE) Four-chamber cardiac magnetic resonance images showing the large apical aneurysm at initial presentation (cine SSFP end-systole; yellow arrows) (A) and corresponding LGE with transmural apical scar (B). Postoperative cine demonstrates complete resolution after aneurysmectomy (green arrows) (C), with a remaining large fibrosis area (D). Cardiac magnetic resonance for the first aneurysm was performed on a 1.5-T Siemens Magnetom Avanto system (syngo MR B17) using an 18-channel cardiac coil. Cine SSFP parameters were TR 53.7 ms, TE 1.57 ms, flip angle 80°, slice thickness 8 mm, matrix 192×192, in-plane resolution 1.46×1.46 mm, and FOV 280 mm. LGE PSIR parameters were TR 939 ms, TE 3.4 ms, TI 360 ms, flip angle 25°, slice thickness 8 mm, matrix 232×256, in-plane resolution 1.09×1.09 mm, and FOV 280×254 mm. Images kindly provided by the Diagnostic Medical Center (CMD) of Marilia, São Paulo, Brazil. LGE = late gadolinium enhancement; PSIR = phase-sensitive inversion recovery; SSFP = steady-state free precession.

Family history was notable for 8 maternal relatives who died in childhood of presumed cardiac causes. His mother had hypertension and diabetes; no known consanguinity.

## Differential Diagnosis

At the time of recurrent arrhythmias and new imaging abnormalities, the differential included.

• Recurrent ischemic aneurysm (unlikely given age and prior angiography)
• Myocarditis or sarcoidosis
• Chagas cardiomyopathy (serology negative)
• Congenital ventricular aneurysm
• Nonischemic cardiomyopathy with regional remodeling (genetic, inflammatory, or idiopathic)

The development of a second, anatomically distinct aneurysm years after complete surgical resolution of the first suggested an underlying diffuse, genetically mediated cardiomyopathy.

## Investigations

### Cardiac magnetic resonance

• 2014 (initial presentation): Cine steady-state free precession (SSFP) showed a large apical aneurysm with marked thinning and systolic dyskinesia. Late gadolinium enhancement (LGE) demonstrated transmural scar confined to the apex. Left ventricular ejection fraction was reduced.
• 2015 (after aneurysmectomy): Cine SSFP showed complete morphological resolution with no residual apical aneurysm. No LGE was present outside the resected region.
• 2020 (recurrence): Cine SSFP revealed a new, large inferolateral basal aneurysm measuring approximately4.5 × 4.7 cm, with thinning, outward bulging, and global left ventricular dilation (Video 3). LGE showed transmural scar confined to the new aneurysmal region, which had been entirely normal in prior imaging.

### Coronary imaging

• 2014: Invasive coronary angiography performed at an outside institution demonstrated normal epicardial coronary arteries (original cine recordings were unavailable for review).
• 2022: Coronary computed tomography angiography showed normal epicardial arteries, supporting a nonischemic etiology (Figure 2, Video 4). Three-dimensional reconstruction also delineated the contour of the inferolateral aneurysm (Video 5).

**Figure 2:**
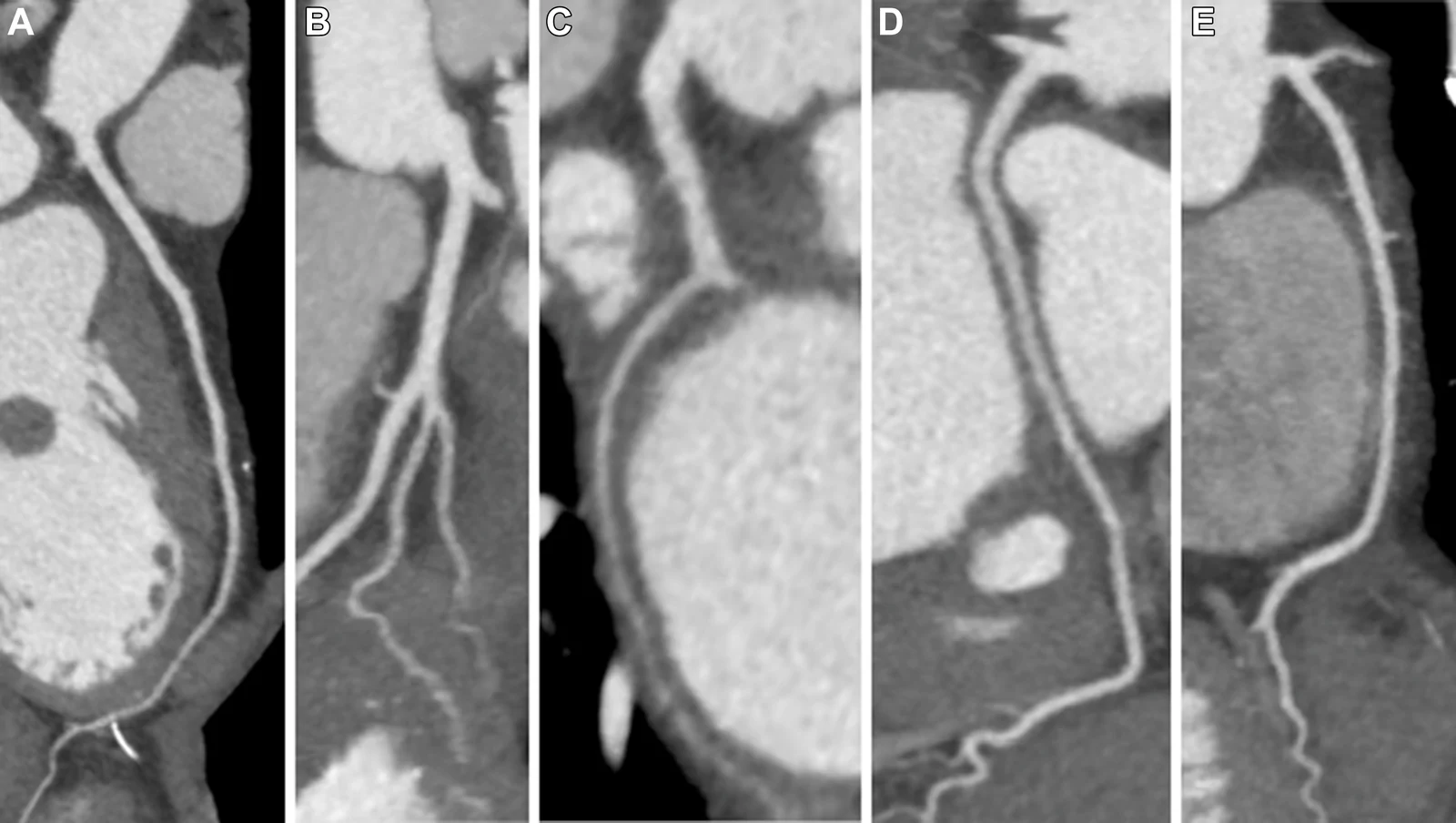
Normal Coronary Arteries Coronary computed tomography angiography demonstrating normal coronary arteries. (A) Left anterior descending artery, (B) first diagonal branch, (C) left circumflex artery and first marginal branch, (D) left circumflex artery and second marginal branch, and (E) right coronary artery and posterior descending artery. Coronary computed tomography angiography was performed on an 80-detector-row Aquilion PRIME scanner (Toshiba/Canon Medical Systems) at 120 kVp and 90 mA, with 0.35-second rotation time, 0.5×80-mm collimation (0.5-mm reconstructed slices), FOV 351 mm, 512×512 matrix, FC03 kernel, and helical acquisition with pitch ≈0.20 and ECG-segmented reconstruction at 75% of the R-R interval (CTDI_vol 8.8 mGy). Iodinated contrast was administered using an arterial bolus-tracking protocol.

### Genetic testing

A targeted cardiomyopathy panel detected a heterozygous *DES* c.785A>T (p.Glu262Val) pathogenic variant. The *DES* gene encodes desmin, an intermediate filament protein essential for sarcomeric alignment and structural integrity. Pathogenic variants in *DES* can lead to myofibrillar disarray and progressive fibrosis.

### Exome sequencing

Exome sequencing revealed 2 variants of unknown significance (VUSs):

• *VCL* c.1226G>A; p.Arg409Gln∗ (cytoskeletal mechanotransduction)
• *CDH2* c.1776G>T; p.Gln592His∗ (N-cadherin; cell–cell adhesion)

Mitochondrial DNA analysis for m.3243A>G was negative.

### Other testing

Routine laboratory evaluation, including Chagas serology, was normal. No systemic inflammatory markers were elevated.

## Management

Given recurrent sustained VT and a new large aneurysm (Figures 3 and 4), the heart team recommended surgical intervention. On January 24, 2023, the patient underwent endoaneurysmorrhaphy with patch repair of the inferolateral aneurysm. Postoperative recovery was uneventful. The operative note described closure of the aneurysm neck with a bovine pericardial patch, followed by closure of the aneurysmal sac over the patch. As no aneurysmal tissue was resected during the procedure. Histopathological confirmation of the lesion was not possible.

**Figure 3:**
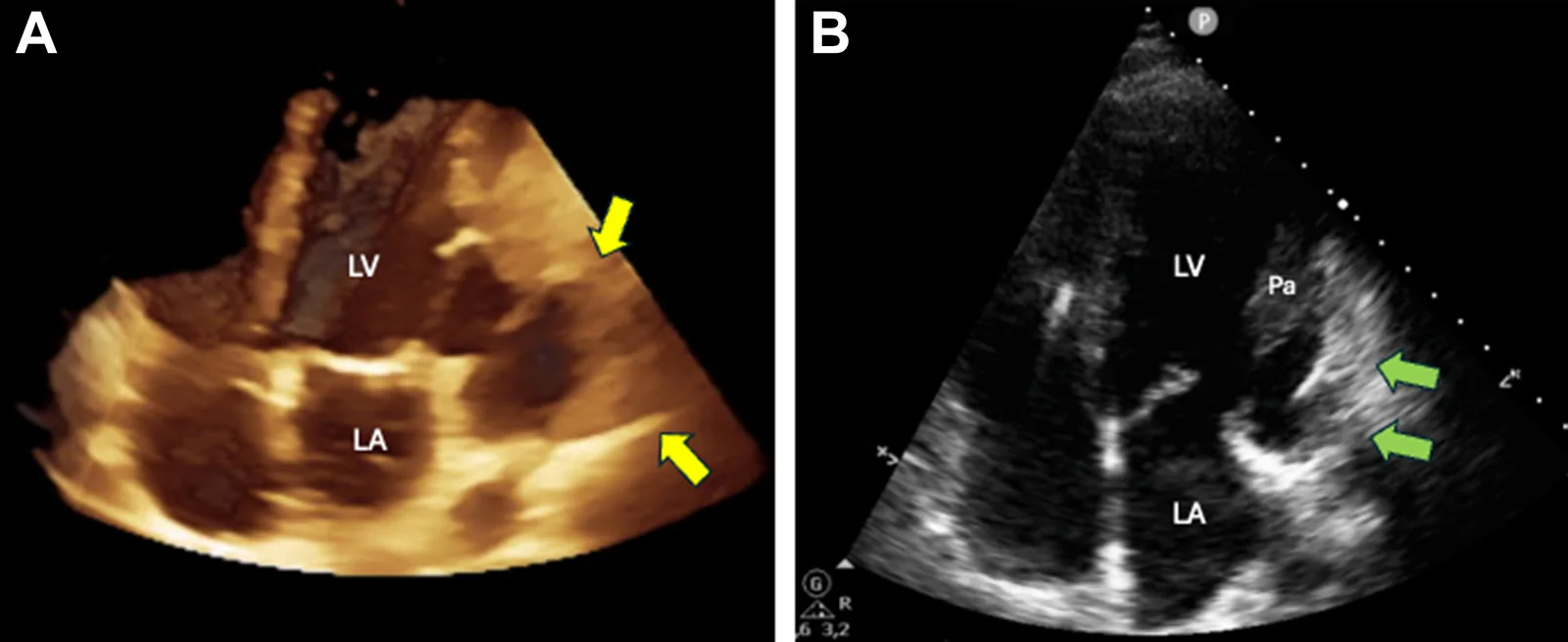
Second Aneurysm (Echocardiographic Evaluation) Transthoracic echocardiographic images demonstrating the inferolateral basal aneurysm before and after surgical repair. (A) Preoperative multiplanar image showing the aneurysm (yellow arrows). (B) Postoperative echocardiographic image demonstrating the repaired aneurysm site (green arrows). Images were acquired using a Vivid E95 system (GE Vingmed Ultrasound; software version 202.71.0). Cine loops were recorded at 26 frames/s during sinus rhythm (heart rate: 59 beats/min). LA = left atrium; LV = left ventricle; Pa = papillary muscle.

**Figure 4:**
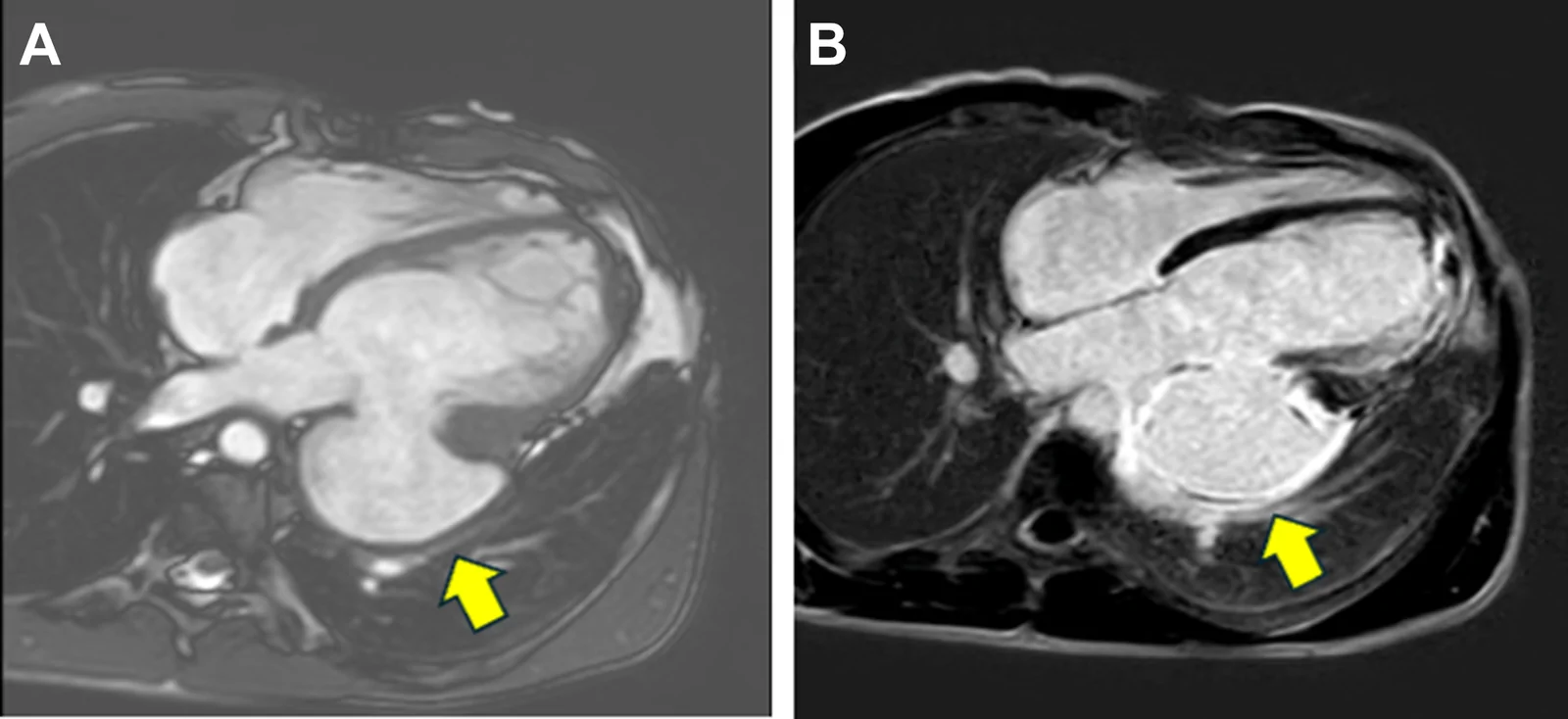
Second Aneurysm (Cine SSFP and LGE) Cardiac magnetic resonance demonstrating the large inferolateral basal aneurysm (yellow arrows) with wall thinning on cine SSFP (A). LGE confirms transmural scar confined to this region (B), previously normal on postoperative studies. Cardiac magnetic resonance for the second aneurysm was performed on a 1.5-T Philips Ingenia system (software 5.7.1) with a multielement cardiac coil. Cine b-TFE parameters were TR 3.48 ms, TE 1.74 ms, flip angle 60°, slice thickness 8 mm, slice spacing 10 mm, matrix 256×256, in-plane resolution 0.98×0.98 mm, and FOV 250 mm. LGE PSIR parameters were TR 6.14 ms, TE 2.99 ms, flip angle 25°, slice thickness 11 mm, slice spacing 11.5 mm, matrix 168×220 (reconstructed 384×384), in-plane resolution 0.91×0.91 mm, and FOV 350 mm. LGE = late gadolinium enhancement; PSIR = phase-sensitive inversion recovery; SSFP = steady-state free precession.

After the aneurysm repair, the patient received an ICD for secondary prevention (Figure 5). Medical therapy included losartan, beta-blocker, amiodarone, anticoagulation, and levothyroxine.

**Figure 5:**
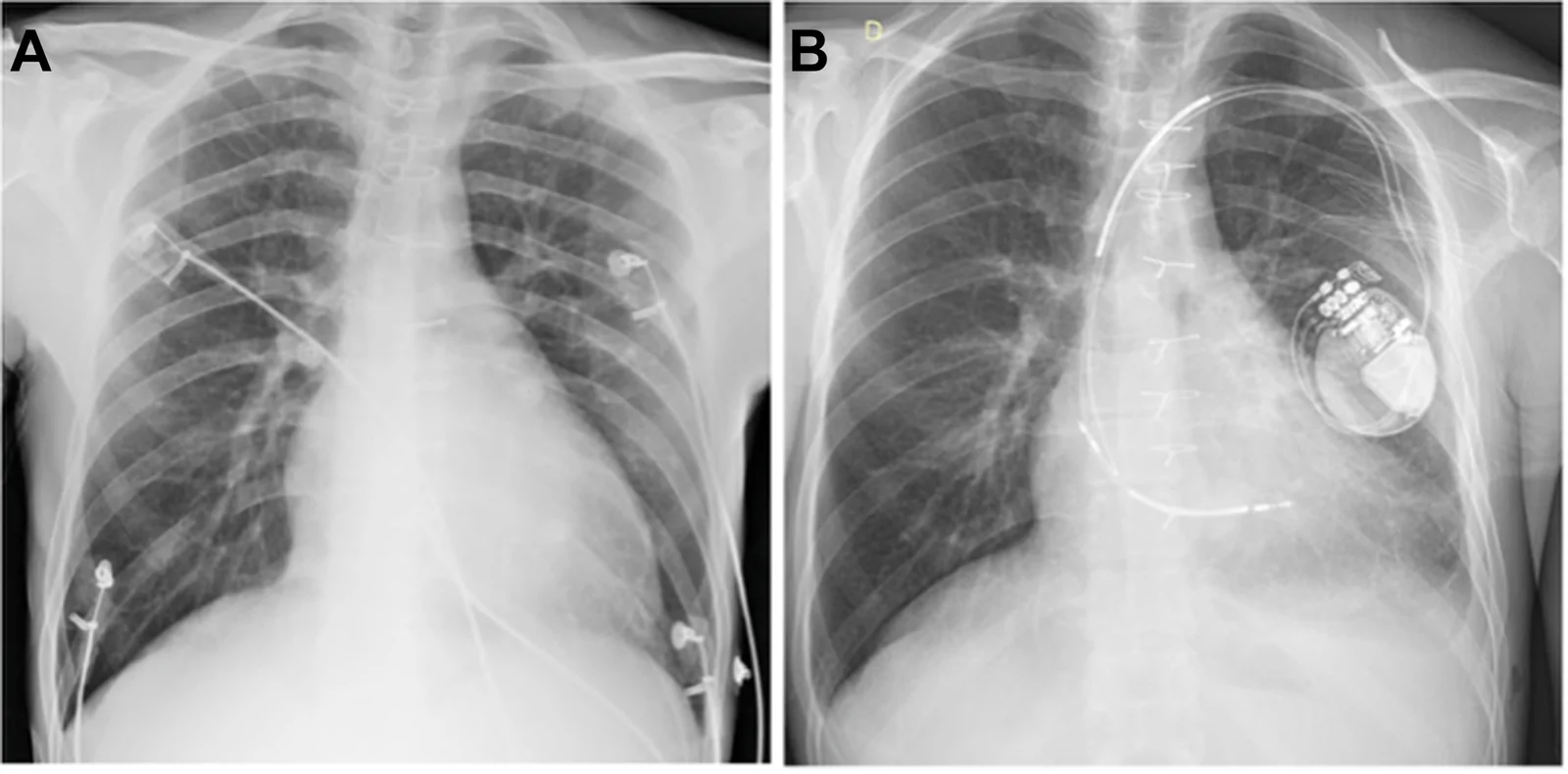
Chest Radiographs Before and After ICD Implantation (A) Anteroposterior chest radiograph while the patient was in the cardiology intensive care unit. (B) Posteroanterior chest radiograph after ICD implantation demonstrating the lead tip positioned at the right ventricular apex. ICD = implantable cardioverter-defibrillator.

## Outcome and Follow-Up

At follow-up, until May 2025, the patient was asymptomatic in NYHA functional class II status. No ICD shocks or sustained ventricular tachyarrhythmias were recorded. Left ventricular function remained moderately reduced, with stable aneurysm repair and no new wall abnormalities.

## Discussion

### Educational value and relevance to clinical practice

This case illustrates several principles directly aligned with guideline-based evaluation of cardiomyopathy and ventricular arrhythmias:

1. CMR is essential in young patients with suspected nonischemic cardiomyopathy.^1^^,^^2^ Americal College of Cardiology/American Heart Association/European Society of Cardiology guidelines highlight CMR as the preferred imaging modality for tissue characterization, scar burden assessment, and evaluation of ventricular aneurysms.^1^^,^^3^^,^^4^
2. Recurrent or anatomically distinct aneurysms in the absence of coronary disease may suggest a genetic etiology. In this patient, CMR uncovered a “migratory” phenotype: Complete postoperative normalization of the apex followed years later by new inferolateral aneurysm in a previously normal region.^3^^,^^5^
3. Transmural LGE confined to new segments provided a structural arrhythmic substrate prompting ICD placement, consistent with guideline recommendations for secondary prevention.^1^^,^^6^

### Genotype-phenotype correlation

Desminopathies typically manifest as cardiomyopathy, skeletal myopathy, conduction disease, or arrhythmias. The p.Glu262Val variant disrupts filament assembly, weakening the myocardium's mechanical resilience under stress.^7^^,^^8^ This mechanism may explain the observed regional weakness in mechanically stressed areas (apex and inferolateral wall), progressive fibrosis with transmural LGE, focal aneurysm formation, and ventricular arrhythmias. This mechanism aligns with:

• regional weakness in mechanically stressed areas (apex and inferolateral wall),
• progressive fibrosis with transmural LGE,
• focal aneurysm formation, and
• ventricular arrhythmias.

The 2 VUSs (*VCL* and *CDH2*) involve proteins at the intercalated disc and may act as modifiers, lowering the threshold for regional failure in a desmin-fragile myocardium.^8^^,^^9^

Although *DES* variants are well described in myofibrillar myopathy and arrhythmogenic phenotypes,^7^^,^^8^ the specific role of the p.Glu262Val variant in focal ventricular aneurysm formation remains uncertain. In this case, the recurrence of nonischemic aneurysms in distinct myocardial regions suggests a possible underlying cytoskeletal predisposition, though a direct causal relationship cannot be established. The 2 additional VUSs (*VCL* and *CDH2*) further complicate interpretation and may represent benign variants, genetic modifiers, or unrelated findings. Thus, the genetic testing results provide supportive evidence but are not definitive, and longitudinal family-based evaluation is warranted for clarification.

### Why this case matters

The sequential development of 2 nonischemic aneurysms in distinct regions, both precisely characterized by CMR, provides a unique illustration of region-dependent myocardial vulnerability in a patient carrying a pathogenic *DES* variant. This case highlights the critical role of CMR in diagnostic reasoning, the importance of integrating genetic testing in cases of unexplained structural heart disease, and the necessity of ICD therapy in the presence of structural scar and sustained VT. It emphasizes:

• the critical role of CMR in diagnostic reasoning,
• the value of integrating genetics in unexplained structural disease, and
• the importance of ICD therapy in the presence of structural scar and sustained VT.

## Conclusions

CMR played a central role in diagnosing sequential nonischemic aneurysms in this young patient. The combination of a compelling family history of early cardiac deaths and a pathogenic *DES* variant suggests that a genetic substrate may contribute to myocardial fragility, although causality cannot be firmly established. This case underscores the importance of multimodality imaging and genetic assessment in unexplained structural cardiomyopathy.

**Visual Summary:**
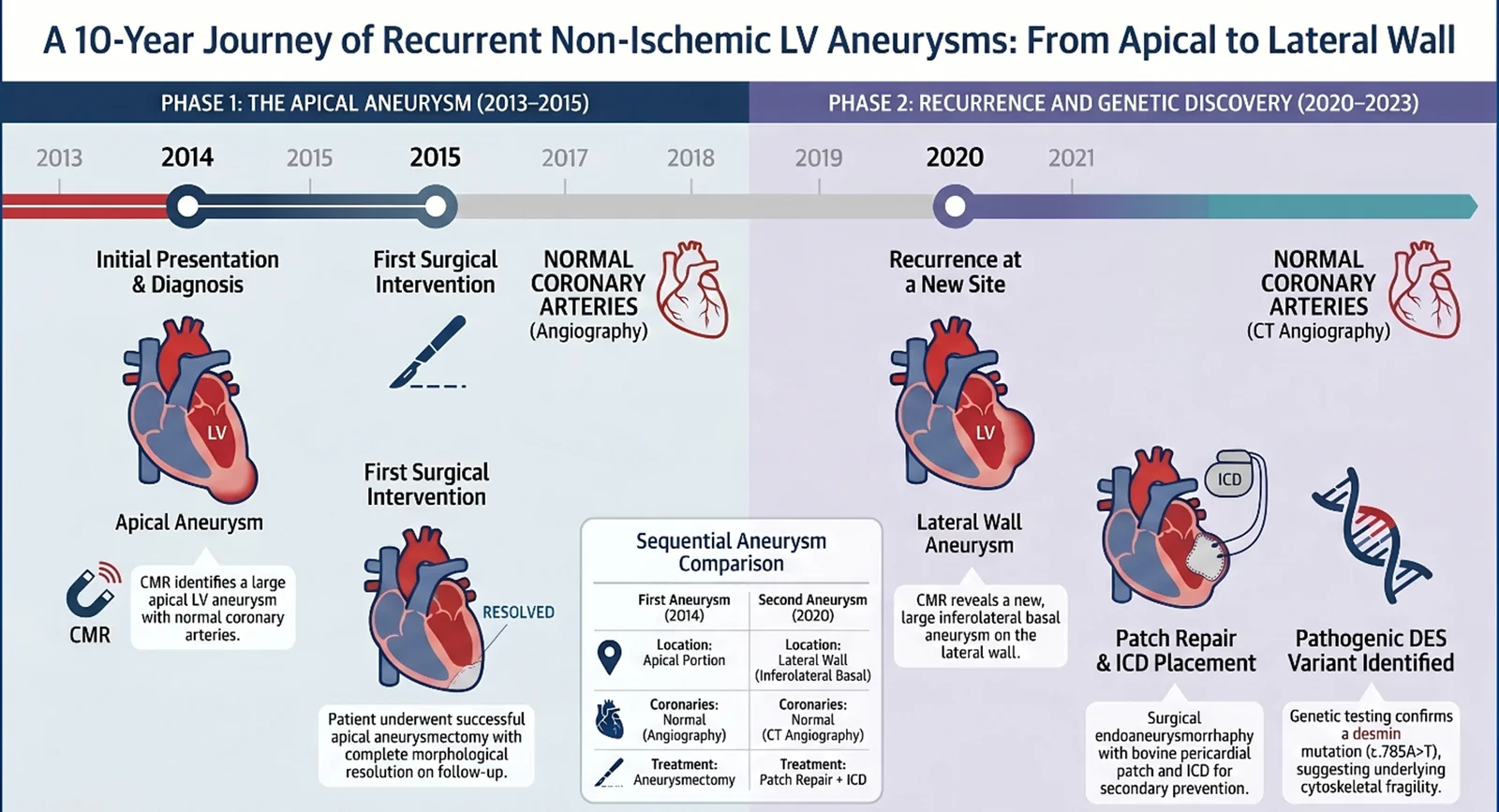
Sequential Development of 2 Anatomically Distinct Nonischemic Left Ventricular Aneurysms in a Patient Carrying a Pathogenic *DES* Variant Initial CMR identified an apical aneurysm with normal coronary arteries on invasive angiography, followed by surgical repair. Five years later, recurrent VT led to the diagnosis of a new inferolateral basal aneurysm on CMR, with normal coronary arteries confirmed by coronary CT angiography. Surgical repair and ICD implantation were performed. CMR = cardiac magnetic resonance; CT = computed tomography; DES = desmin; ICD = implantable cardioverter-defibrillator; LV = left ventricle; VT = ventricular tachycardia.

## Funding Support and Author Disclosures

The authors have reported that they have no relationships relevant to the contents of this paper to disclose.

**Take-Home Messages**

• Cardiac magnetic resonance is crucial in identifying sequential, anatomically distinct left ventricular aneurysms.
• The combination of recurrent nonischemic aneurysms and a pathogenic *DES* variant suggests a possible cytoskeletal genetic cardiomyopathy with arrhythmic risk.

## Appendix

Video 1 Cine SSFP Showing the Initial Apical Aneurysm Specifications: 1.5-T Siemens Magnetom Avanto system (syngo MR B17) using an 18-channel cardiac coil. Cine SSFP parameters were TR 53.7 ms, TE 1.57 ms, flip angle 80°, slice thickness 8 mm, matrix 192 × 192, in-plane resolution 1.46 × 1.46 mm, and FOV 280 mm. Images kindly provided by the Diagnostic Medical Center (CMD) of Marilia, São Paulo, Brazil.

Video 2 Cine SSFP Postoperative Images Confirming Resolution of the Apical Aneurysm Specifications: 1.5-T Siemens Magnetom Avanto system (syngo MR B17) using an 18-channel cardiac coil. Cine SSFP parameters were TR 53.7 ms, TE 1.57 ms, flip angle 80°, slice thickness 8 mm, matrix 192×192, in-plane resolution 1.46 × 1.46 mm, and FOV 280 mm. Images kindly provided by the Diagnostic Medical Center (CMD) of Marilia, São Paulo, Brazil.

Video 3 Cine SSFP Showing the Second Inferolateral Aneurysm Specifications: 1.5-T Philips Ingenia system (software 5.7.1) with a multielement cardiac coil. Cine b-TFE parameters were TR 3.48 ms, TE 1.74 ms, flip angle 60°, slice thickness 8 mm, slice spacing 10 mm, matrix 256×256, in-plane resolution 0.98 × 0.98 mm, and FOV 250 mm.

Video 4 Coronary CT Angiography Demonstrating Normal Coronary Arteries and Inferolateral Aneurysmal Contour Coronary computed tomography angiography was performed on an 80-detector-row Aquilion PRIME scanner (Toshiba/Canon Medical Systems) at 120 kVp and 90 mA, with 0.35-second rotation time, 0.5×80-mm collimation (0.5-mm reconstructed slices), FOV 351 mm, 512×512 matrix, FC03 kernel, and helical acquisition with pitch ≈0.20 and ECG-segmented reconstruction at 75% of the R-R interval (CTDI_vol 8.8 mGy). Iodinated contrast was administered using an arterial bolus-tracking protocol.

Video 5 Three-Dimensional Reconstruction of the Coronary CT Angiography Showing the Second Inferolateral Aneurysm (Yellow Arrows) Coronary computed tomography angiography was performed on an 80-detector-row Aquilion PRIME scanner (Toshiba/Canon Medical Systems) at 120 kVp and 90 mA, with 0.35-second rotation time, 0.5×80-mm collimation (0.5-mm reconstructed slices), FOV 351 mm, 512×512 matrix, FC03 kernel, and helical acquisition with pitch ≈0.20 and ECG-segmented reconstruction at 75% of the R-R interval (CTDI_vol 8.8 mGy). Iodinated contrast was administered using an arterial bolus-tracking protocol.
